# Supplementary material for: MetaQTL: a package of new computational methods for the meta-analysis of QTL mapping experiments
Source: BMC Bioinformatics. 2007 Feb 8;8:49. doi: 10.1186/1471-2105-8-49 (PMC1808479; doi:10.1186/1471-2105-8-49)
Supplement: Additional File 7 — MetaQTL Package : jar file and tutorial. This Zip archive contains both the MetaQTL JAR file and the files of the tutorial. [file 1471-2105-8-49-S7.zip › org.inra.metaqtl/doc/org/metaqtl/bio/entity/factory/OntologyTabFactory.html]

OntologyTabFactory


|  |  |  |
| --- | --- | --- |
| **Overview** \| **Package** \| **Class** \| **Use** \| **Tree** \| **Deprecated** \| **Index** \| **Help** | |  |
| **PREV CLASS**   **NEXT CLASS** | **FRAMES**    **NO FRAMES**     **All Classes** |  |
| SUMMARY: NESTED \| FIELD \| CONSTR \| METHOD | DETAIL: FIELD \| CONSTR \| METHOD |  |


---


## org.metaqtl.bio.entity.factory Class OntologyTabFactory

```
java.lang.Object
  org.metaqtl.bio.entity.factory.BioEntityFactory
      org.metaqtl.bio.entity.factory.OntologyTabFactory
```

---

``` public class OntologyTabFactory extends BioEntityFactory ```

A factory to deal with `Ontology`.

---

| **Field Summary** | |
| --- | --- |
| `static java.lang.String[]` | `HEADER` |


**Constructor Summary**

`OntologyTabFactory()`


| **Method Summary** | |
| --- | --- |
| `IBioEntity` | `load(java.io.InputStream inputStream)` |
| `IBioEntity` | `load(java.io.Reader reader)`              Term\_id : the term identifient. |
| `void` | `unload(IBioEntity entity, java.io.OutputStream stream)` |
| `void` | `unload(IBioEntity entity, java.io.Writer writer)` |

**Methods inherited from class java.lang.Object**

`clone, equals, finalize, getClass, hashCode, notify, notifyAll, toString, wait, wait, wait`

**Field Detail**

### HEADER

```
public static final java.lang.String[] HEADER
```


**Constructor Detail**

### OntologyTabFactory

```
public OntologyTabFactory()
```


**Method Detail**

### load

```
public IBioEntity load(java.io.InputStream inputStream)
                throws java.io.IOException
```

:   **Specified by:**: `load` in class `BioEntityFactory`
:   **Throws:**: `java.io.IOException`

---


### load

```
public IBioEntity load(java.io.Reader reader)
                throws java.io.IOException
```

:   - Term\_id : the term identifient.
    - Term\_name : the term name
    - Parent\_id : the term parent id
    - Synonyms : the synonyms
    - Definition : the definition of the term

    :   **Specified by:**: `load` in class `BioEntityFactory`
    :   **Throws:**: `java.io.IOException`

---


### unload

```
public void unload(IBioEntity entity,
                   java.io.Writer writer)
            throws java.io.IOException
```

:   **Specified by:**: `unload` in class `BioEntityFactory`
:   **Throws:**: `java.io.IOException`

---


### unload

```
public void unload(IBioEntity entity,
                   java.io.OutputStream stream)
            throws java.io.IOException
```

:   **Specified by:**: `unload` in class `BioEntityFactory`
:   **Throws:**: `java.io.IOException`


---


|  |  |  |
| --- | --- | --- |
| **Overview** \| **Package** \| **Class** \| **Use** \| **Tree** \| **Deprecated** \| **Index** \| **Help** | |  |
| **PREV CLASS**   **NEXT CLASS** | **FRAMES**    **NO FRAMES**     **All Classes** |  |
| SUMMARY: NESTED \| FIELD \| CONSTR \| METHOD | DETAIL: FIELD \| CONSTR \| METHOD |  |


---
